# MRI-based radiomics and ADC values are related to recurrence of endometrial carcinoma: a preliminary analysis

**DOI:** 10.1186/s12885-021-08988-x

**Published:** 2021-11-24

**Authors:** Kaiyue Zhang, Yu Zhang, Xin Fang, Jiangning Dong, Liting Qian

**Affiliations:** 1 Department of Radiation Oncology, Anhui Provincial Hospital Affiliated to Anhui Medical University, Hefei, 230001 China; 2 Department of Radiology, First Affiliated Hospital of University of Science and Technology of China, Anhui Provincial Cancer Hospital, Hefei, 230031 China; 3 Department of Radiation Oncology, First Affiliated Hospital of University of Science and Technology of China, Hefei, 230001 China

**Keywords:** Endometrial neoplasms, Recurrence, Risk factors, Apparent diffusion coefficient, Radiomics

## Abstract

**Background:**

To identify predictive value of apparent diffusion coefficient (ADC) values and magnetic resonance imaging (MRI)-based radiomics for all recurrences in patients with endometrial carcinoma (EC).

**Methods:**

One hundred and seventy-four EC patients who were treated with operation and followed up in our institution were retrospectively reviewed, and the patients were divided into training and test group. Baseline clinicopathological features and mean ADC (ADC_mean_), minimum ADC (ADC_min_), and maximum ADC (ADC_max)_ were analyzed. Radiomic parameters were extracted on T2 weighted images and screened by logistic regression, and then a radiomics signature was developed to calculate the radiomic score (radscore). In training group, Kaplan–Meier analysis was performed and a Cox regression model was used to evaluate the correlation between clinicopathological features, ADC values and radscore with recurrence, and verified in the test group.

**Results:**

ADC_mean_ showed inverse correlation with recurrence, while radscore was positively associated with recurrence. In univariate analyses, FIGO stage, pathological types, myometrial invasion, ADC_mean_, ADC_min_ and radscore were associated with recurrence. In the training group, multivariate Cox analysis showed that pathological types, ADC_mean_ and radscore were independent risk factors for recurrence, which were verified in the test group.

**Conclusions:**

ADC_mean_ value and radscore were independent predictors of recurrence of EC, which can supplement prognostic information in addition to clinicopathological information and provide basis for individualized treatment and follow-up plan.

**Supplementary Information:**

The online version contains supplementary material available at 10.1186/s12885-021-08988-x.

## Background

Endometrial carcinoma (EC) is the sixth most commonly diagnosed cancer in women. With the increasing prevalence of risk factors such as physical inactivity and overweight, the incidence of endometrial cancer continues to rise [1, 2]. Surgery is the main method for the initial treatment of EC including laparoscopic or robotic resection of uterus, cervix, fallopian tube and ovary, and sentinel lymph node assessment [3]. Although most endometrial cancers can be diagnosed at an early stage and have 5-year survival rate of over 90%, recurrence and final mortality approximately occur in 20% of endometrioid carcinoma (type I) and 50% of non-endometrioid carcinoma (type II) [4–6]. Women with recurrent or metastatic diseases have 5-year survival rates as low as 17–55% [7, 8]. Unfortunately, little progress has been made in improving the survival rate of EC in the past decades. Therefore, early identification of risk factors for recurrence is an important challenge to improve the prognosis of EC patients.

Clinicopathological factors, such as FIGO stage, pathological type and muscular invasion, etc. are common prognostic factors that affect the formulation of surgical plan, but they can only be accurately evaluated after surgery [9]. Due to the limitations of specimen collection, preoperative endometrial biopsy may be difficult to fully reflect the characteristics and heterogeneity of the tumor. Therefore, it is necessary to develop an early, comprehensive and non-invasive evaluation method to evaluate the possible adverse prognosis of EC. MRI is an important tool to preliminarily assess the extent of EC lesions. The apparent diffusion coefficient (ADC) of diffusion-weighted imaging (DWI) can reflect the malignancy of the tumor, which has been proved to be valuable in the diagnosis, typing and grade of EC [10–12]. Gharibvand et al. [11]. reported that DWI sequence (ADC value) had high accuracy in diagnosing benign endometrial lesions and differentiating malignant lesions. Yashar et al. [12]. conducted a mate-analysis involving 11 studies and found that ADC values had good diagnostic accuracy in differentiating EC from benign lesions, with combined sensitivity and specificity of 93%(87~96%; I^2^ = 41.19%) and 94% (88% ~ 97%; I^2^ = 46.91%). In addition, jiang et al.’s study [13] showed that ADC value of grade 1 EC patients was significantly higher than that of grade 3 patients, and ADC value of patients with high expression of Ki-67 was significantly lower than that of patients with low expression of Ki-67. These studies suggest that ADC value has the potential to predict EC biological behavior, but its role in the prognostic assessment of EC remains unclear.

Radiomics mines pixel distribution features from radiological images and transforms them into quantitative data, reflecting the heterogeneity within tumors [14]. Radiomic parameters derived from MRI, CT and PET-CT have been suggested as effective tools for diagnosis, risk assessment or treatment response of malignant tumors [15–17]. The nomogram of radiomics extracted from ADC sequence can be used to predict EC histological classification before operation [18]. Rodríguez-Ortega et al. [19] combined texture parameters extracted from T2 weighted images (T2WI) and ADC sequences with semi-quantitative parameters of DWI and dynamic contrast-enhanced (DCE) for preoperative evaluation of myometrial invasion. A multicenter study established a model for evaluating pelvic lymph node status based on radiomics of MRI, which helped radiologists improve diagnostic efficiency [20]. In addition, Wang et al. [21] found that the radiomic feature of GLCMEntropy extracted from PET/CT for tumors is a potential predictor of PD1 expression in EC. The purpose of this study is to explore whether postoperative recurrence of patients with EC can be reflected in MRI-based ADC value and radiomics.

## Methods

### Patient population

This retrospective study was approved by the institutional review board and the informed consent was waived. Patients with EC who underwent 3.0 MRI before treatment in our institution from January 2015 to December 2019 were included retrospectively. Inclusion criteria are as follows: (a) pathologically confirmed EC; (b) available clinical and postoperative pathological data; (c) MRI was performed within 1 month before surgery; (d) surgical treatment and follow-up were performed in our hospital. Exclusion criteria are as follows: (a) diagnosis of malignant tumor other than endometrial cancer (*n* = 4); (b) distant metastasis occurred at the time of diagnosis (IVB stage) (*n* = 5); (c) tumors were invisible on MRI or MRI with serious motion artifact (*n* = 24); (d) incomplete pathological report or medical record (*n* = 14); (e) patients lost to follow-up (*n* = 20). Finally, a total of 174 patients diagnosed with EC were identified. The last follow-up was in March 2021. All patients were randomly divided into the training group and the test group in a ratio of 3:2.

### MR imaging

All patients underwent pelvic MRI examination before operation using a standard imaging protocol. MRI was performed on a 3.0 T MRI system (Signa Excite HD, GE, Milwaukee, WI, USA) using an 8-channel body coil. Prior to MRI examinations, patients fasted for at least 4 h and were given intramuscular 15 mg shyoscine butylbromide half an hour before examination. During image acquisition, the patient remained supine with a semi-filled bladder.

Scanning sequence and parameters: (1) Axial T1-weighted images (T1WI): a field of view (FOV): 38 cm × 26 cm, repetition time (TR): 500 ms, echo time (TE): 7.2 ms, slice thickness: 6 mm, inter-slice gap: 2 mm, matrix size: 352 × 192. (2) Axial and axial fast spin-echo (FSE) T2WI: FOV: 24 cm × 24 cm, TR: 4600 ms, TE: 68 ms, slice thickness: 3 mm, inter-slice gap: 1 mm, matrix size: 320 × 256; (3) Oblique sagittal T2WI: FOV: 26 cm × 24 cm, TR: 4600 ms, TE: 68 ms, slice thickness: 6 mm, inter-slice gap: 2 mm, matrix size: 320 × 256; (4) Axial DWI: FOV: 38 cm × 26 cm, TR: 4000 ms, TE: 65 ms, slice thickness: 4 mm, inter-slice gap: 1 mm, matrix size: 96 × 130, b-value: 0 and 1000 mm^2^/s. Contrast agent GD-DTPA (Magnevist, Bayer Schering, Berlin, Germany) was injected through the anterior cubital vein with a high pressure syringe at a flow rate of 2.5 ml/s at 0.1 mmol/kg. After injection of contrast agent, 3 phase axial and 1 phase delayed sagittal scanning were started 25 s later.

### Histologic and pathologic diagnosis

All surgical specimens were examined and reported by gynecologic pathologists. Tumor staging were performed according to standard 2018 FIGO criteria. Myometrial invasion, lymphovascular space invasion (LVSI) and lymph node metastasis (LNM) were confirmed under microscope according to the corresponding diagnostic criteria. Ki-67 was detected by immunohistochemistry (streptavidin-peroxidase method). Ki-67 positive is defined as obvious brownish yellow granules in the cytoplasm of tumor cells, and the staining intensity is higher than the nonspecific staining background. Under 200x field of vision, 10 fields were randomly selected, and the average positive percentage of each field was defined as the proliferation index (PI).

Postoperative follow-up of patients: 3 to 6 months for the first 2–3 years, 6 months until 5 years, and then annually, any time when there are related symptoms such as vaginal bleeding, abdominal distension and pain. Surveillance included gynecological examination, imaging and pathological biopsy if necessary.

### Image analysis

**Fig. 1:**
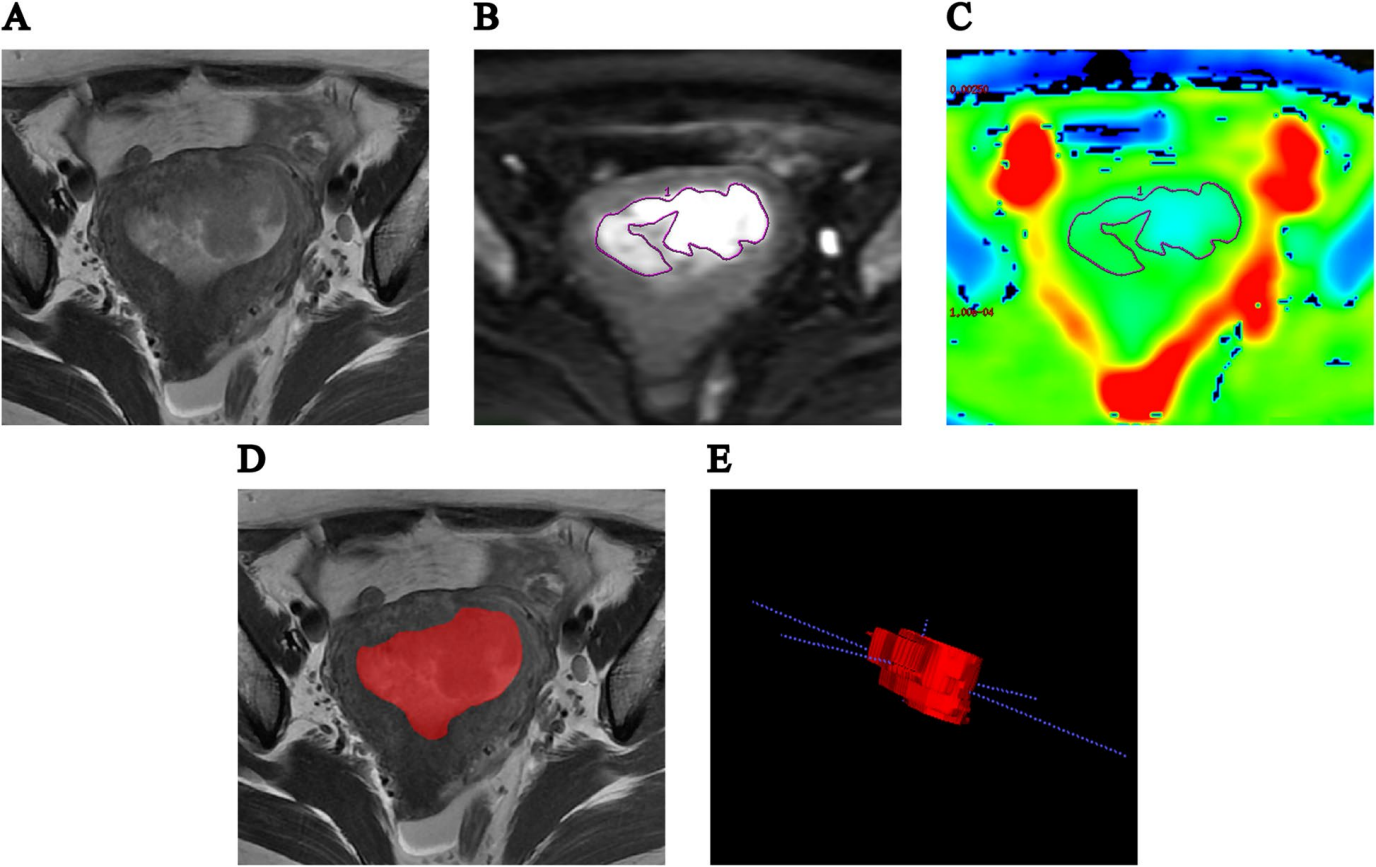
MR images of a 52-yearold woman with endometrioid carcinoma of stage IA. **A** Axial T2-weighted image demonstrated a mass in the uterine cavity, approximately 5.9 cm × 4.0 cm in size. **B** DWI (b = 1000 s/ mm^2^) showed the method of placing ROI within the tumor area. **C** ADC image showed that the ADC_mean_, ADC_min_ and ADC_max_ value were 0.710 × 10^− 3^, 0.673 × 10^− 3^ and 0.914 × 10^− 3^ mm^2^/s. **D** T2-weighted image showed the ROI of tumor segmentation. **E** The three-dimensional volume of interest

The images were assessed by two radiologists with 8 and 10 years (reader 1 and reader 2) of professional experience in pelvic MRI independently. Moreover, they were both blinded to each other’s results, and pathological and clinical data. Tumor was defined as a mass with signal hyperintensity on DWI and hypointensity on ADC map, compared with the signal of surrounding adjacent tissues. Meanwhile, T2WI sequence was referenced. ADC related parameters measurements of the tumor were executed on an ADC map using GE Advantage Workstation 4.6, Function Tool software. The region of interest (ROI) was manually delineated in the slice containing the largest tumor layer, including the largest tumor area as much as possible and avoiding bleeding and necrotic tissue (Fig. 1.A − C). Each reader measured ADC values for each patient three times, the mean ADC (ADC_mean_), minimum ADC (ADC_min_), and maximum ADC (ADC_max_) were recorded, respectively, and three average values were calculated.

Tumor segmentation were performed on axial T2WI sequence using ITK-SNAP (Version 3.6.0, http://www.itksnap.org) software by reader 2. ROIs were sketched manually on all MRI levels containing tumor, and the ROIs covered the whole tumor as much as possible. Then the ROIs of all layers were fused to get the three-dimensional volume of interest (VOI). Finally, radiomic parameters were extract from VOIs using AK (Analysis Kit, Kinetics Version 2.1, GE Healthcare) software, following the IBSI standards, with a total of 1130 (Fig. 1.D − E). More information about radiomic feature extraction methods is provided in Additional file 1: Supplementary Methods 1 and Additional file 2. To investigate the stability of radiomic features extracted by different readers and the same reader, 30 patients were randomly selected for tumor segmentation 2 weeks later.

### Statistical analysis

The data were analyzed with SPSS v. 26.0 (Chicago, IL, USA) and R (Version 3.6.1, http://www.r-project.org). For continuous variables (including age, ADC_mean_, ADC_min,_ ADC_max_, tumor size and radscore), the data of normal distribution were represented by mean ± standard deviation (SD) and analyzed by independent sample *t* test; the data of non-normally distribution were represented by Median (interquartile range) and analyzed by Mann–Whitney U-test in the nonparametric rank sum test. Categorical variables (FIGO stage, pathological types, myometrial invasion, LVSI, LNM and Ki-67) were expressed using constituent ratios and analyzed by Chi-square test or fisher’s exact test. The intraclass correlation coefficient (ICC) was used to evaluate the intra-observer and inter-observer consistency of ADC_mean_, ADC_min_, ADC_max_ values and radiomic parameters. The ADC_mean_, ADC_min_, ADC_max_ values having higher intra-observer ICC were retained. The radiomic features with intra-observer and inter-observer ICC > 0.75 were considered stable and were retained for subsequent analyses. Logistic regression analysis was used to select radiomic parameters in training group. Each patient’s radscore was calculated based on the regression coefficient of the selected radiomic features using multi-factor linear weighting in the training group. Receiver operating characteristic (ROC) curve and Youden index were used to obtain cut-off values of ADC_mean_, ADC_min_, ADC_max_ value and radscore in training group. In the training group, Spearman’s bivariate correlation test was used to analyze the correlation between ADC_mean_, ADC_min_, ADC_max_ value, radscore and recurrence, the Kaplan–Meier method was used to calculate survival rate and draw survival curve, Log–rank method was used for univariate analysis. Variables with *P* < 0.05 were included in a multivariate Cox regression model and independent prognostic factors of survival were identified with *P* < 0.05. The association of these factors with DFS was then verified in the test group, patients were divided into high-risk group and low-risk group according to the risk factors, Kaplan-Meier curves were used to analyze DFS of high-risk and low-risk groups and Log–rank method was used to compare the differences.

## Results

### Patient characteristics and outcomes

A total of 174 patients with EC treated in our institution were included, 162 patients underwent laparoscopic surgery and 12 patients had open laparotomy. Among all the patients, 48 patients received postoperative radiotherapy, and 105 patients received adjuvant chemotherapy or concurrent chemotherapy.

The median follow-up of 174 patients was 31 months (range, 4–69 months). The median follow-up time of recurrent cohort was 18 months (range, 4–50 months). Tumor recurrence was recorded in a total of 27 (15.5%) patients of the 174 cases. There were 3 (11.1%) isolated pelvis recurrences, 1 (3.7%) isolated vaginal recurrence, 6 (22.2%) isolated abdominal failure, 6 (22.2%) combined pelvic and distant failure, and 11 (40.8%) distant failure. The 3-year and 5-year disease-free survival (DFS) of the entire cohort were 91.2% (95%CI: 86.9–95.5%) and 75.2% (95%CI: 64.8–85.6%).

The comparison of clinicopathological features between non-recurrent and recurrent cohorts of training and test group is shown in Table 1. The onset age of recurrent group was significantly higher than that of non-recurrent group (60.1 ± 11.5 vs 55.6 ± 8.5, *P* = 0.018). Based on median, tumor size was divided into ≥3.5 cm group and < 3.5 cm group, and Ki-67 PI was divided into ≥50% and < 50% group. There was a significant difference in FIGO staging between recurrence group and non-recurrence group (*P* < 0.001). The pathological types were 137 cases of endometrioid carcinoma and 37 cases of non-endometrioid carcinoma, and there were more recurrences in patients with non-endometrioid carcinoma compared to those with endometrioid carcinoma (37.8% vs 9.5%, *P* < 0.001). Pathological evaluation showed that 27.6% of all patients included had deep myometrial invasion (≥50%), and there were more recurrences in patients with myometrial invasion ≥50% compared to those with < 50% (31.2% vs 9.5%, P < 0.001). The recurrence rate of LVSI present patients was significantly higher than that of LVSI absent patients (37.9% vs 11.0%, P < 0.001). However, there was no significant difference between the recurrence and no-recurrence groups with regards to tumor size LNM and Ki-67 PI.

**Table 1:** Clinicopathological characteristics of recurrent and non-recurrent endometrial carcinoma in training and test group

Characteristics	Training group (***n*** = 104)		***P*** value	Test group (***n*** = 70)		***P*** value	***P*** value
	No recurrence (***n*** = 88)	Recurrence (***n*** = 16)		No recurrence (***n*** = 59)	Recurrence (***n*** = 11)		
**Age at diagnosis (yr)**	55.0 ± 9.2	59.6 ± 13.7	0.035	56.6 ± 7.2	60.9 ± 7.9	0.280	0.154
**FIGO stage**			< 0.001			0.006	0.026
IA	44(50.0%)	4(25.0%)		44(74.6%)	5(45.4%)		
IB	12(13.6%)	2(12.5%)		3(5.1%)	2(18.2%)		
II	18(20.5%)	1(6.2%)		11(18.6%)	1(9.1%)		
IIIA	4(4.5%)	3(18.8%)		0(0.0%)	1(9.1%)		
IIIC1	7(8.0%)	2(12.5%)		0(0.0%)	1(9.1%)		
IIIC2	3(3.4%)	1(6.2%)		1(1.7%)	1(9.1%)		
IVA	0(0.0%)	3(18.8%)		0(0.0%)	0(0.0%)		
**Tumor size (cm)**			0.035			0.750	0.442
< 3.5	42(47.7%)	5(31.3%)		31(52.5%)	5(45.5%)		
≥3.5	46(52.3%)	11(68.7%)		28(47.5%)	6(54.5%)		
**Pathological types**			0.078			< 0.001	0.454
Endometrioid carcinoma	74(84.1%)	10(62.5%)		50(84.7%)	3(27.3%)		
Non-endometrioid carcinoma	14(15.9%)	6(37.5%)		9(15.3%)	8(72.7%)		
**Myometrial invasion**			0.001			0.135	0.730
< 50%	68(77.3%)	6(37.5%)		46(78.0%)	6(54.5%)		
≥50%	20(22.7%)	10((62.5%)		13(22.0%)	5(45.5%)		
**LVSI**			0.001			0.143	0.305
Absent	76(86.4%)	8(50.0%)		53(89.8%)	8(72.7%)		
Present	12(13.6%)	8(50.0%)		6(10.2%)	3(27.3%)		
**LNM**			0.410			0.062	0.106
Absent	78(88.6%)	13(81.3%)		58(98.3%)	9(81.8%)		
Present	10(11.4%)	3(18.7%)		1(1.7%)	2(18.2%)		
**Ki-67**			1.00			0.515	0.878
< 50%	44(50.0%)	8(50.0%)		30(50.9%)	4(36.4%)		
≥50%	44(50.0%)	8(50.0%)		29(49.1%)	7(63.6%)		

*LVSI* lymphovascular space invasion, *LNM* lymph node metastasis

### Extraction of ADC value and radiomic features

A total of 1130 radiomic features were obtained from axial T2WI sequence, of which 112 features had both intra-observer and inter-observer ICC > 0.75. Through multivariate logistic regression, 2 radiomic features were obtained as independent discriminant features in the training group, which were glcm_DifferenceEntropy and lbp-3D-m1_firstorder_MeanAbsoluteDeviation (Additional file 1: Supplementary Table S 1–2), then the radiomics score (radscore) of each patient was calculated as a new variable, according to the linear combination of regression coefficients (Additional file 1: Supplementary Methods 2). In the training group, the radscore of recurrence patients was significantly higher than that of non-recurrence patients, and the radscore was positively correlated with recurrence. ADC_mean_ in recurrence group was significantly lower than that in non-recurrence group, and was negatively correlated with recurrence (Table 2).

**Table 2:** ROC curve of ADC values and radscore in training group

	NO recurrence	Recurrence	***p*** value	AUC	Cut-off value	Sensitivity (%)	Specificity (%)	Correlation with recurrence status (rs)
ADC_mean_	0.867(0.183)	0.761(0.184)	0.008	0.709	0.795	78.4	62.5	−0.272
ADC_min_	0.700(0.185)	0.680(0.142)	0.123	0.611	0.743	38.6	87.5	−0.152
ADC_max_	1.196(0.375)	1.077(0.331)	0.092	0.633	1.154	53.4	68.7	−0.162
Radscore	−2.154 ± 1.059	−1.051 ± 0.702	< 0.001	0.820	−1.227	68.8	85.2	0.368

*ADC* apparent diffusion coefficient, *AUC* area under curve

**Fig. 2:**
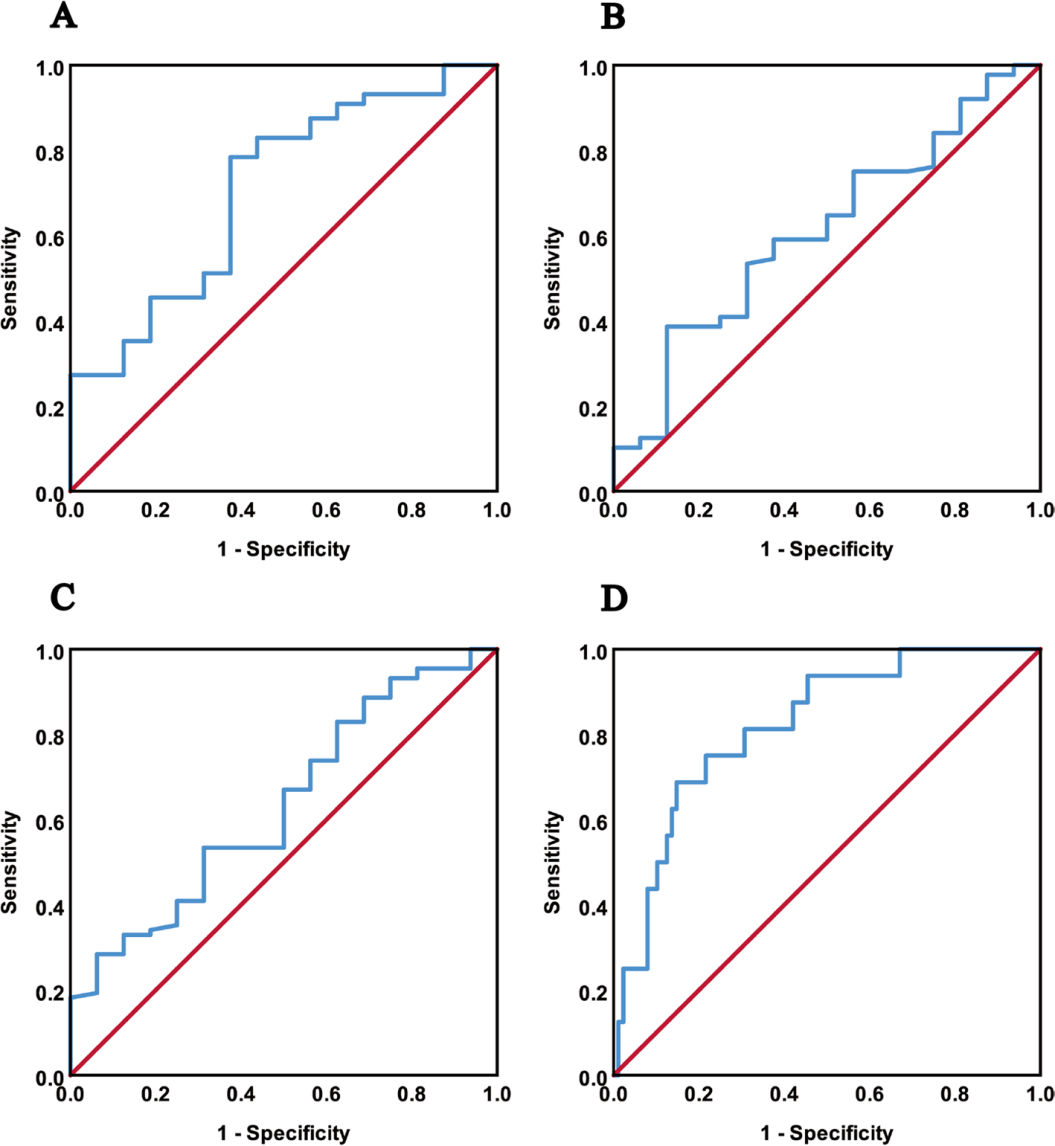
ROC curve analysis of ADC_mean_, ADC_min_, ADC_max_ and radscore predicting recurrence in training group. **A** ADC_mean_ predicting recurrence, AUC = 0.709, **B** ADC_min_ predicting recurrence, AUC = 0.611, **C** ADC_max_ predicting recurrence, AUC = 0.633, and **D** radscore predicting recurrence, AUC = 0.820

ROC curve results of ADC_mean_, ADC_min_, ADC_max_ and radscore for predicting recurrence in training group are shown in Table 2. The cut-off values of ADC_mean_, ADC_min_, ADC_max_ and radscore were 0.795, 0.743, 1.154 and − 1.227, according to Youden index. The ROCs of ADC_mean_, ADC_min_, ADC_max_ and radscore in training group are shown in Fig. 2.

The ADC_min_ value of Ki-67 ≥ 50% group was significantly lower than that of Ki-67 < 50% group (*P* < 0.05). There was no significant difference in the ADC_mean_ and ADC_max_ value between the two groups (Table 3).

**Table 3:** Comparison of ADC values between Ki-67 ≥ 50% group and Ki-67 < 50% group in training group

	Ki-67 < 50% group	Ki-67 ≥ 50% group	***Z*** value	***P*** value	Correlation with Ki-67
ADC_mean_	0.864 (0.175)	0.859 (0.201)	−1.07	0.286	−0.221
ADC_min_	0.706 (0.111)	0.674 (0.208)	−2.15	0.032	−0.131
ADC_max_	1.196 (0.330)	1.083 (0.362)	−1.02	0.309	−0.126

*ADC* apparent diffusion coefficient

### Univariate and multivariate analysis of factors associated with recurrence

**Fig. 3:**
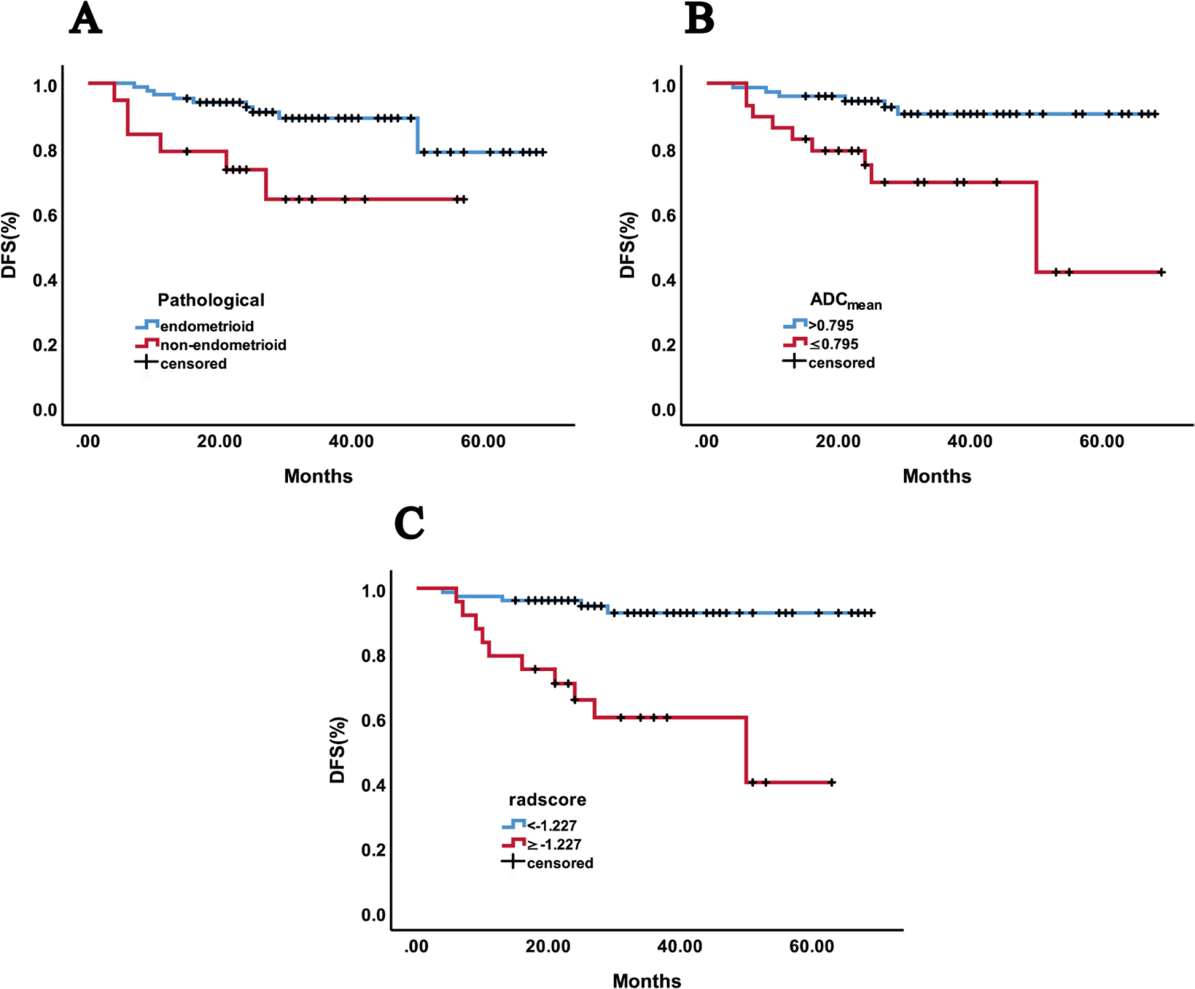
DFS curves of patients with EC in training group. **A**: endometrioid vs. non-endometrioid carcinoma, *P* = 0.01, **B**: ADC_mean_ > 0.795 vs. ≤ 0.795, *P* = 0.001, and **C**: radscore < − 1.227 vs. ≥ − 1.227, *P* < 0.001

**Fig. 4:**
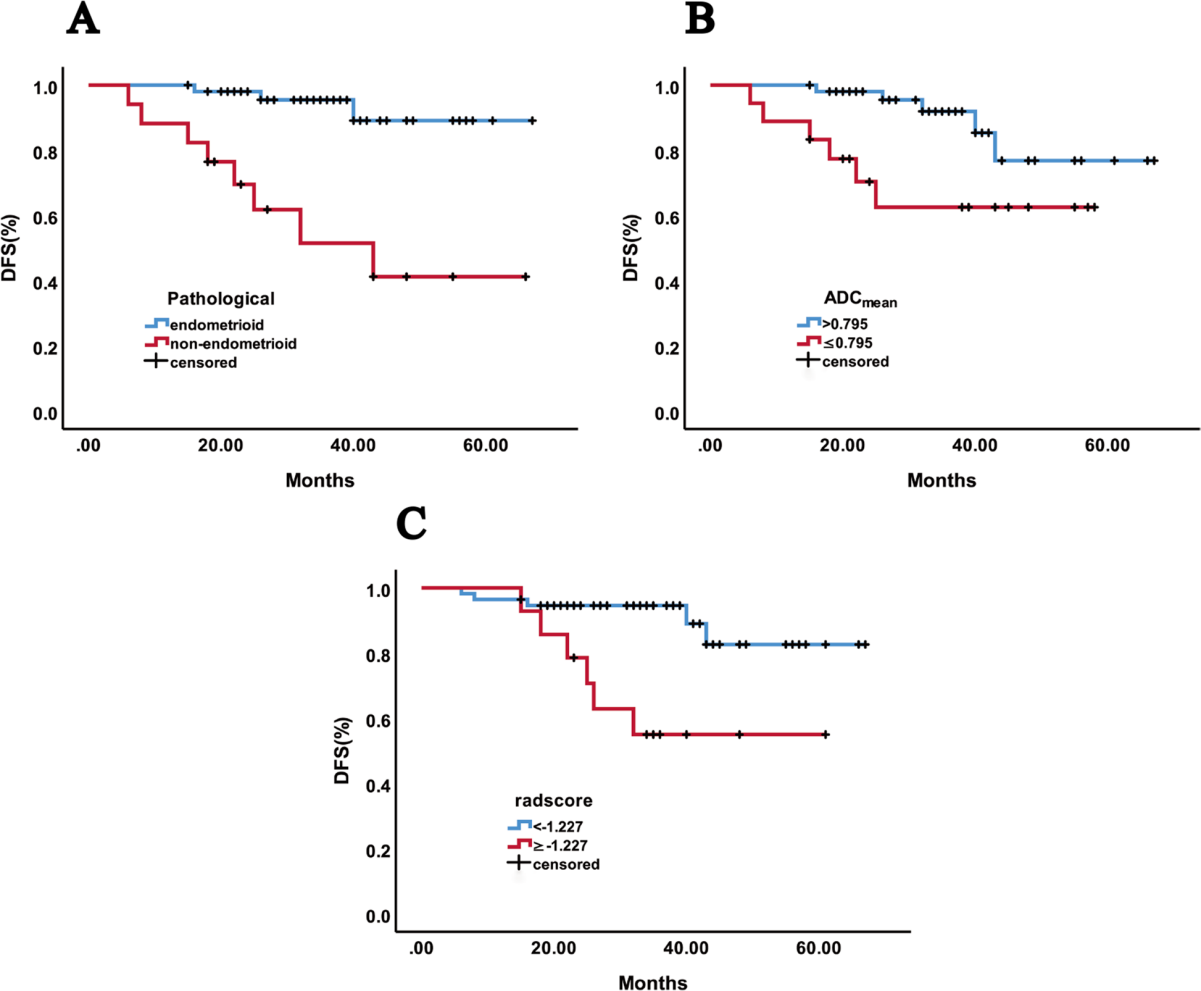
DFS curves of patients with EC in test group. **A**: endometrioid vs. non-endometrioid carcinoma, *P* < 0.001, **B**: ADC_mean_ >0.795 vs. ≤0.795, *P* = 0.017, and **C**: radscore < − 1.227 vs. ≥ − 1.227, *P* = 0.003

Univariate analysis revealed that six factors were predictive of recurrence; FIGO stage (hazard ratio [HR] = 5.15; 95% CI = 1.92–13.86; *P* < 0.001), pathological types (HR = 3.49; 95% CI = 1.26–9.65; *P* = 0.01), depth of muscular invasion (HR = 2.97; 95% CI = 1.07–8.23; *P* = 0.027), ADC_mean_ (HR = 4.98; 95% CI = 1.81–13.74; P = 0.001), ADC_min_ (HR = 4.48; 95% CI = 1.02–19.79; *P* = 0.029) and radscore (HR = 7.88; 95% CI = 2.73–22.78;*P* < 0.001) were significantly correlated with disease-free survival (DFS). There was no association between the risk of recurrence and age, tumor size, LVSI, LNM, ADC_max_ and Ki-67 (Table 4). In multivariate analysis, non-endometrioid (HR = 3.90; 95% CI = 1.27–12.02; *P* = 0.018), ADC_mean_ (HR = 3.57; 95% CI = 1.02–12.44; *P* = 0.046) and radscore (HR = 5.05; 95% CI = 1.60–16.00; *P* = 0.006) were independent predictors of recurrence (Table 5) (Fig. 3). When patients in the test group were stratified at risk using pathological type and ADC_mean_ and radscore cutoff values derived from the training set, respectively, the survival curves of the two risk groups were significantly different (for pathological type, *P* < 0.001; for ADC_mean_, *P* = 0.017; for radscore *P* = 0.003) (Fig. 4).

**Table 4:** Univariate analysis of recurrence factors for DFS of endometrial carcinoma in training group

Predictive factors	Recurrence		
	HR	95%CI	***p***-value
Age at diagnosis	1.61	0.58–4.45	0.537
FIGO stage (I–II vs. III–IV)	5.15	1.92–13.86	< 0.001
Tumor size (< 3.5 cm vs. ≥3.5 cm)	1.79	0.62–5.14	0.274
Pathological types (endometrioid vs. non-endometrioid)	3.49	1.26–9.65	0.01
Myometrial invasion (< 50% vs. ≥50%)	2.97	1.07–8.23	0.027
LVSI (absent vs. present)	0.53	0.12–2.33	0.388
LNM (absent vs. present)	1.49	0.42–5.23	0.529
Ki-67 (< 50% vs. ≥50%)	0.91	0.34–2.46	0.857
ADC_mean_ (> 0. 795 vs. ≤0.795)	4.98	1.81–13.74	0.001
ADC_min_ (> 0.743 vs. ≤0.743)	4.48	1.02–19.79	0.029
ADC_max_ (> 1.154 vs. ≤1.154)	2.16	0.75–6.23	0.142
Radscore (<−1.227 vs. ≥ − 1.227)	7.88	2.73–22.78	< 0.001

*LVSI* lymphovascular space invasion, *LNM* lymph node metastasis, *ADC* apparent diffusion coefficient, *HR* hazard ratio, *CI* confidence interval

**Table 5:** Multivariate analysis of recurrence factors for DFS of endometrial carcinoma in training group

Predictive factors	Recurrence		
	HR	95%CI	***P*** value
FIGO stage (I–II vs. III–IV)	1.74	0.54–5.56	0.351
Pathological types (endometrioid vs. non-endometrioid)	3.90	1.27–12.02	0.018
Myometrial invasion (< 50% vs. ≥50%)	1.52	0.51–4.55	0.452
ADC_mean_ (> 0. 795 vs. ≤0.795)	3.57	1.02–12.44	0.046
ADC_min_ (> 0.743 vs. ≤0.743)	1.16	0.19–6.97	0.872
Radscore (<−1.227 vs. ≥ − 1.227)	5.05	1.60–16.00	0.006

*ADC* apparent diffusion coefficient, *HR* hazard ratio, *CI* confidence interval

## Discussion

Traditionally, prognostic factors for EC include surgical stage, histological factors [22, 23], and molecular factors such as P53 mutation [24]. This information is often only available from histological evaluation after surgery, so preoperative risk assessment of recurrence is limited. As a routine preoperative imaging examination of EC, MRI can provide anatomical information, and DWI and radiomics analysis may reveal the prognostic information of EC. In addition to clinical and pathological factors, we also investigated the correlation between ADC values (including ADC_mean_, ADC_min_, ADC_max_) and radscore and recurrence in this study. We identified risk factors associated with recurrence and reduced survival of EC after surgery, including FIGO stage, pathologic type, depth of muscular invasion, ADC_mean_, ADC_min_ and radscore. Non-endometrioid cancer, ADC_mean_ and radscore were independent predictors of EC recurrence, which can be used as clinically relevant tumor markers in preoperative risk stratification and prognosis assessment of EC.

Although the number of recurrent patients is small in our study, we identified the clinical and pathological risk factors related to recurrence of EC patients including FIGO stage, pathological type, and myometrial invasion depth. These factors have been associated with recurrence or poor outcome in EC in other studies. Women who were initially diagnosed with advanced disease (FIGO III- IV) had a higher risk of recurrence and were more likely to develop extrapervic metastases [8]. Patients with low-grade (grade 1-2) endometrioid cancer (type I) often have better outcomes than those with high-grade (grade 3) endometrioid cancer and non-endometrioid cancer (type II) [25]. A study exploring recurrence factors for stage I endometrioid adenocarcinoma observed that large tumor size, and muscular invasion were the most important predictors of recurrence [9]. The study of Bosse et al. [22] confirmed that substantial LVSI was the strongest prognostic factor for recurrence and metastasis and overall survival of EC.

Notably, we did not find age of onset, tumor size, LVSI or LNM as factors related to the increased risk of recurrence. The subjects of our study were concentrated in the 50-60 years old, which was a homogeneous population, with few patients under 50 years old or over 70 years old. In our study, the tumor size was defined as the maximum diameter of the tumor, which was often the extent to which tumor tissue invaded the endometrium rather than the depth. So, there was no significant difference in tumor size between the relapsed and non-relapsed groups, and tumor size had no significant effect on survival. Less cases with positive lymph node metastasis and LVSI may be the reason why LNM and LSVI had no effect on survival time.

In recent years, many studies have demonstrated the value of MRI in predicting histopathological features of gynecological tumors. Satta et al. [26] prospectively analyzed the association between preoperative MRI imaging parameters and histopathology in 44 EC patients, and found that the quantitative parameters of preoperative DWI, intravoxel incoherent motion (IVIM) and dynamic contrast-enhanced (DCE) MRI can reflect the physiological and microscopic characteristics of EC. Chen et al. [10] reported that type II EC had lower ADC values, and ADC values were important when identifying type II and type I ECs. The study of Jiang et al. [13] indicated that the ADC values of high grade, stage IB and high Ki-67 expression patients were significantly lower than those of low grade, stage IA and low Ki-67 expression patients with EC. Zhang et al. [27] reported that lower ADC were observed in tumor with deep myometrial invasion and LVSI than tumor without deep myometrial invasion and LVSI. Similarly, our previous studies [28] explored the role of ADC values in EC histological features and demonstrated that lower ADC values were associated with higher grade, non- endometrioid subtype and a higher risk of deep muscular-layer invasion and lymph node metastasis. These findings suggest a potential association between ADC related parameters and adverse outcomes in EC. Our present study found that lower ADC_mean_ and ADC_min_ value were related to the recurrence of EC and ADC_mean_ was shown to be an independent predictor of DFS in multivariate analysis. In addition, we also found that ADC_min_ was significantly negatively correlated with Ki-67 expression level. Many studies have explored the correlation between ADC related parameters and histological molecules in different tumors. Ma et al. [29] reported that the Ki-67 PI, HIF-1α expression and VEGF expression in prostate cancer were correlated inversely with ADC. Surov et al. [30] conducted a multicenter study of breast cancer and found that ADC values were significantly correlated with Ki-67 expression in breast cancer. Similarly, Zhang et al. [31] reported that lower mean ADC values were associated with higher Ki-67 expression in Type I ovarian epithelial cancer. ADC values can reflect the diffusion degree of water molecules and is closely related to cell density. Yan et al. [20] and Reyes-Pérez et al. [32] explained that the decrease of ADC can act as markers for tumor’s high cellularity, proliferation, perfusion and less extracellular space, which means higher tumor load, tumor residue and recurrence. Therefore, DWI and ADC values may be noninvasive techniques for evaluating endometrial cancer cell proliferation So, it is not difficult to explain the correlation between ADC and prognosis and recurrence.

Heterogeneity is an important characteristic of malignant tumors and the basis of tumor recurrence and metastasis [33, 34]. Radiomics can extract features that cannot be observed by naked eyes and convert them into data information to quantify the heterogeneity in the image [35, 36]. Radiomics derived from MRI have been proposed as a reliable tool for accurate diagnosis and risk assessment in several cancer types, e.g. in cervical [37], brain [38], and breast [39]. Similarly, a study has reported that radiomics analysis based on MRI was an effective tool for preoperative risk stratification in EC [40]. In this context, MRI-based radiomics analysis was available to predict recurrence in patients with EC, and 2 radiomic parameters were selected to predict recurrence. Among these, Glcm_DifferenceEntropy was a measure of the randomness/variability in neighborhood intensity value differences, lbp features were the observation of the whole tumor range and the extraction of high-dimensional features. The radscore was calculated according to the combination of regression coefficients of each parameter, which was an independent predictor of relapse in both univariate and multivariate analyses. Patients were grouped into risk groups based on radscore, and patients with higher radscore had worse DFS. Sigmund et al. [41]. reported that thirteen MRI-derived tumor radiomic parameters significantly predicted reduced recurrence- and progression-free survival in univariable Cox regression analysis, and T1c_Kurtosis2 was the top-ranked prognostic texture parameter independently predicted reduced survival. The study of Yan et al. [16] manifested that MRI-based radiomics achieved high diagnostic performance for predicting LVSI of EC preoperatively, and was helpful for early identification of poor prognosis. In addition, the whole-tumor radiomic features were found to significantly predict progression-free survival at hazard ratios of 4.6–9.8 in the research of Fasmer et al. [42], albeit in a small sample size. Although the sample size, MRI sequence, and radiomic parameter extraction methods were different in the previous study, we cannot deny that radiomics and texture analysis may mine more prognostic information than clinical factors, and can be used as a biomarker to assist clinical practice.

Our current study expanded the sample size compared with other previous studies and simultaneously explored the correlation between clinicopathological factors, DWI quantitative parameters, and radiomics and EC recurrence. There are some limitations in our research. This is a single-center study, which needs to be further verified by a large multi-center database. The absence of stratification of staging and pathologic types may influence the estimation of survival and recurrence outcomes. A short follow-up time and less recurrence cases may limit the predictive value of the covariates.

## Conclusions

In conclusion, EC patients are at certain risk of recurrence, and early identification of risk factors for recurrence and enhanced treatment intensity are significant to improve prognosis. ADC_mean_ values and MRI-derived radiomics may provide additional prognostic information in addition to traditional prognostic factors.

## Supplementary Information

**Additional file 1: Supplementary Method S1**. Radiomic features. **Supplementary Methods 2**. Calculation Formula for the radiomics score (radscore). **Supplementary Table S1**. Multivariate logistic regression analysis of radiomic parameters in training group. **Supplementary Table S2**. ICCs of intra-observer and inter-observer of ADC and selected radiomic parameters.

**Additional file 2: Supplementary Material Table 1**: IBSI Reporting guidelines.

## Data Availability

Not applicable.

## Abbreviations

ADC: Apparent diffusion coefficient
MRI: Magnetic resonance imaging
EC: Endometrial carcinoma
radscore: Radiomic score
LVSI: Lymphovascular space invasion
DWI: diffusion-weighted imaging
T1WI: T1-weighted images
FOV: Field of view
TR: Repetition time
TE: Echo time
FSE: Fast spin-echo
LNM: Lymph node metastasis
ROI: Region of interest
VOI: Volume of interest
ICC: Intraclass correlation coefficient
ROC: Receiver operating characteristic
DFS: Disease-free survival
AUC: Area under curve
