# Supplementary material for: MRI-based radiomics and ADC values are related to recurrence of endometrial carcinoma: a preliminary analysis
Source: BMC Cancer. 2021 Nov 24;21:1266. doi: 10.1186/s12885-021-08988-x (PMC8611883; doi:10.1186/s12885-021-08988-x)
Supplement: Supplementary file 1 — Additional file 1: Supplementary Method S1. Radiomic features. Supplementary Methods 2. Calculation Formula for the radiomics score (radscore). Supplementary Table S1. Multivariate logistic regression analysis of radiomic parameters in training group. Supplementary Table S2. ICCs of intra-observer and inter-observer of ADC and selected radiomic parameters. [file 12885_2021_8988_MOESM1_ESM.docx]

**Supplementary Method S1. Radiomic features.**

In this study, a total of 1130 radiomic features were extracted from axial T2WI, including 18 First-order statistics features, 14 Morphological features and 1098 texture features.

1. The First-order statistics features based on the gray level histogram describe the

distribution of MRI voxel intensities.

1.1 10^th^ percentile 1.2 90^th^ percentile 1.3 Energy 1.4 Entropy 1.5 Interquartile range

1.6 Kurtosis 1.7 Maximum 1.8 Mean 1.9 Mean absolute deviation 1.10 Median

1.11 Minimum 1.12 Range 1.13 Robust mean absolute deviation

1.14 Root mean square 1.15 skewness 1.16 Total energy 1.17 Uniform 1.18 Variance

2. Morphological features describe the geometric features of ROI, such as area and volume, and filters are not required.

2.1 Elongaton 2.2 Flatness 2.3 Least axis length 2.4 Major axis length

2.5 Volume (mesh) 2.6 Minor axis length 2.7 Sphericity 2.8 Surface area

2.9 Surface to volume ratio 2.10 Volume (voxel counting)

2.11 Maximum 2D diameter (Column) 2.12 Maximum 2D diameter (Row)

2.13 Maximum 2D diameter (Slice) 2.14 Maximum 3D diameter

3. Texture features reflect the surface structure of the tumor surface with slow or

periodic changes, expressed by the gray level distribution of the neighborhood of the

voxel.

3.1 The Grey Level Co-occurrence Matrix (GLCM) is a matrix that expresses how combinations of discretised intensities (grey levels) of neighbouring pixels, or voxels in a 3D volume, are distributed along one of the image directions. The (i,j)^th^ element of this matrix represents the number of times the combination of levels ii and jj occur in two pixels in the image, that are separated by a distance of δδ pixels along angle θθ. The distance δδ from the center voxel is defined as the distance according to the infinity norm.

3.1.1 Autocorrelation 3.1.2 Joint Average 3.1.3 Cluster Prominence

3.1.4 Cluster Shade 3.1.5 Cluster Tendency 3.1.6 Contrast 3.1.7 Correlation

3.1.8 Difference Average 3.1.9 Difference Entropy 3.1.10 Difference Variance

3.1.11 Joint Energy 3.1.12 Joint Entropy 3.1.13 IMC1 3.1.14 ICM2

3.1.15 IDM 3.1.16 MCC 3.1.17 IDMN 3.1.18 ID 3.1.19 IND 3.1.20 Inverse Variance

3.1.21 Maximum Probability 3.1.22 Sum Average 3.1.23 Sum Entropy

3.1.24 Sum of Squares

3.2 The gray level dependence matrix (GLDM) quantifies gray level dependencies in an image. A gray level dependency is defined as the number of connected voxels within distance δδ that are dependent on the center voxel. A neighboring voxel with gray level jj is considered dependent on center voxel with gray level ii if |i−j|≤α|i−j|≤α. In a gray level dependence matrix P(i,j) the (i,j)^th^ element describes the number of times a voxel with gray level ii with jj dependent voxels in its neighborhood appears in image.

3.2.1SDE 3.2.2 LDE 3.2.3 GLN 3.2.4 DN 3.2.5 DNN 3.2.6 GLV 3.2.7 DV 3.2.8 DE 3.2.9 LGLE 3.2.10 HGLE 3.2.11 SDLGLE 3.2.12 SDHGLE

3.2.13 LDLGLE 3.2.14 LDHGLE

3.3 The gray level run length matrix (GLRLM) quantifies gray level runs, which are defined as the length in number of pixels, of consecutive pixels that have the same gray level value. In a gray level run length matrix P(i,j|θ), the (i,j)^th^ element describes the number of runs with gray level ii and length jj occur in the image (ROI) along angle θθ.

3.3.1SRE 3.3.2LRE 3.3.3GLN 3.3.4GLNN 3.3.5RLN 3.3.6 RLNN 3.3.7 RP

3.3.8 GLV 3.3.9 RV 3.3.10 RE 3.3.11 LGLRE 3.3.12 HGLRE 3.3.13 SRLGLE

3.3.14 SRHGLE 3.3.15 LRLGLE 3.3.16 LRHGLE

3.4 The gray level size zone (GLSZM) is defined as the number of connected voxels that share the same gray level intensity. A voxel is considered connected if the distance is 1 according to the infinity norm (26-connected region in a 3D, 8-connected region in 2D). In a gray level size zone matrix P(i,j) the (i,j)^th^ element equals the number of zones with gray level ii and size jj appear in image.

3.4.1 SAE 3.4.2 LAE 3.4.3 GLN 3.4.4 GLNN 3.4.5 SZN 3.4.6 SZNN 3.4.7 ZP

3.4.8 GLV 3.4.9 ZV 3.4.10 ZE 3.4.11 LGLZE 3.4.12 HGLZE 3.4.13 SALGLE

3.4.14 SAHGLE 3.4.15 LALGLE 3.3.4.16 LAHGLE

3.5 The neighbourhood grey tone difference matrix (NGTDM) corresponds contains the sum of grey level differences of pixels/voxels with discretised grey level i and the average discretised grey level of neighbouring pixels/voxels within a Chebyshev distance δ.

3.5.1 Contrast 3.5.2 busyness 3.5.3 Complexity 3.5.4 Strength 3.5.5 Coarseness

3.6 The Local binary patterns are methods for image feature classification in computer vision. Firstly, the detection window is divided into 16×16 small areas (cells). 8 points in its ring neighborhood (or multiple points in ring neighborhood) are compared clockwise or counterclockwise. If the central pixel value is larger than the neighboring point, the neighboring point is assigned 1; otherwise, 0 is assigned. The histogram of each cell is then calculated, that is, the frequency of occurrence of each number (presumably a decimal number) (that is, a binary sequence of statistics on whether each pixel point is larger than the neighborhood point), and the histogram is normalized.

$${LBP}_{P,R}=\sum_{p=0}^{p-1} s\left( g_{p}-g_{c} \right)2^{p}$$

$$s\left( x \right)=\left\{ \begin{aligned} 1,x\geq0 \\ 0,x<0 \end{aligned} \right.$$

3.7 Wavelet transform feature is to decompose the image at multiple levels, and carry out wavelet transform on the sub-band image after each level of classification, and construct the feature vector of texture through the transform coefficient. Common coefficients such as: Mean, Entropy, Standard Deviation, Energy

**Supplementary Methods 2 Calculation Formula for the radiomics score (radscore)**

Radscore = -2.0598-0.9089 * glcm_DifferenceEntropy+0.5651 * lbp-3D-m1_firstorder_MeanAbsoluteDeviation

**Supplementary Table S1 Multivariate logistic regression analysis of radiomic parameters in training group**

| **Variable** | **OR** | **95% CI** | ***p* value** |
| --- | --- | --- | --- |
| glcm_DifferenceEntropy | 0.403 | 0.234–0.695 | 0.001 |
| lbp-3D-m1_firstorder_MeanAbsoluteDeviation | 1.760 | 1.033–2.998 | 0.038 |

**Supplementary Table S2 ICCs of intra-observer and inter-observer of ADC and selected radiomic parameters**

| **Variable** | intra-observer **ICC** | inter-observer **ICC** |
| --- | --- | --- |
| glcm_DifferenceEntropy | 0.975 | 0.897 |
| lbp-3D-m1_firstorder_MeanAbsoluteDeviation | 0.992 | 0.969 |
| ADC_mean_ | 0.946 | 0.772 |
| ADC_min_ | 0.862 | 0.828 |
| ADC_max_ | 0.963 | 0.785 |
