# Supplementary material for: MRI-based radiomics and ADC values are related to recurrence of endometrial carcinoma: a preliminary analysis
Source: BMC Cancer. 2021 Nov 24;21:1266. doi: 10.1186/s12885-021-08988-x (PMC8611883; doi:10.1186/s12885-021-08988-x)
Supplement: Supplementary file 2 — Additional file 2: Supplementary Material Table 1: IBSI Reporting guidelines. [file 12885_2021_8988_MOESM2_ESM.docx]

**Supplementary Material Table 1: IBSI Reporting guidelines**

| Section and Topic | Modality | Item | Description | Page^*^/ Description |
| --- | --- | --- | --- | --- |
| Patient |  |  |  |  |
| Region of interest |  | 1 | Describe the region of interest that is being imaged | Page 6 |
| Patient preparation |  | 2a | Describe specific instructions given to patients prior to image acquisition. | Page 6 |
|  |  | 2b | Describe administration of drugs to the patient prior to image acquisition | Page 6 |
|  |  | 2c | Describe the use of specific equipment for patient comfort during scanning | Page 6 |
| Radioactive tracer | PET, SPECT | 3a | Describe which radioactive tracer was administered to the patient, | NA |
|  | PET, SPECT | 3b | Describe the administration method. | NA |
|  | PET, SPECT | 3c | Describe the injected activity of the radioactive tracer at administration. | NA |
|  | PET, SPECT | 3d | Describe the uptake time prior to image acquisition. | NA |
|  | PET, SPECT | 3e | Describe how competing substance levels were controlled | NA |
| Contrast agent |  | 4a | Describe which contrast agent was administered to the patient. | Page 6 |
|  |  | 4d | Describe the administration method. | Page 6 |
|  |  | 4c | Describe the injected quantity of contrast agent. | Page 6 |
|  |  | 4d | Describe the uptake time prior to image acquisition. | Page 6 |
|  |  | 4e | Describe how competing substance levels were controlled. | NA |
| Comorbidities |  | 5 | Describe if the patients have comorbidities that affect imaging | Page 6 |
| Acquisition |  |  |  |  |
| Acquisition protocol |  | 6 | Describe whether a standard imaging protocol was used, and where its description may be found. | Page 6 |
| Scanner type |  | 7 | Describe the scanner type(s) and vendor(s) used in the study. | Page 6 |
| Imaging modality |  | 8 | Clearly state the imaging modality that was used in the study | Page 6 |
| Static/dynamic scans |  | 9a | State if the scans were static or dynamic. | Page 6 |
|  | Dynamic scans | 9b | Describe the acquisition time per time frame. | Page 6 |
|  | Dynamic scans | 9c | Describe any temporal modelling technique that was used. | NA |
| Scanner calibration |  | 10 | Describe how and when the scanner was calibrated. | NA |
| Patient instructions |  | 11 | Describe specific instructions given to the patient during acquisition | Page 6 |
| Anatomical motion correction |  | 12 | Describe the method used to minimise the effect of anatomical motion. | Page 6 |
| Scan duration |  | 13 | Describe the duration of the complete scan or the time per bed position. | NA |
| Tube voltage | CT | 14 | Describe the peak kilo voltage output of the X-ray source. | NA |
| Tube current | CT | 15 | Describe the tube current in mA. | NA |
| Time-of-flight | PET | 16 | State if scanner time-of-flight capabilities are used during acquisition. | NA |
| RF coil | MRI | 17 | Describe what kind RF coil used for acquisition. | Page 6 |
| Scanning sequence | MRI | 18a | Describe which scanning sequence was acquired | Page 6 |
|  | MRI | 18b | Describe which sequence variant was acquired. | Page 6 |
|  | MRI | 18c | Describe which scan options apply to the current sequence | Page 6 |
| Repetition time | MRI | 19 | Describe the time in ms between subsequent pulse sequences. | Page 6 |
| Echo time | MRI | 20 | Describe the echo time in ms | Page 6 |
| Echo train length | MRI | 21 | Describe the number of lines in k-space that are acquired per excitation pulse. | NA |
| Inversion time | MRI | 22 | Describe the time in ms between the middle of the inverting RF pulse to the middle of the excitation pulse. | NA |
| Flip angle | MRI | 23 | Describe the flip angle produced by the RF pulses. | NA |
| Acquisition type | MRI | 24 | Describe the acquisition type of the MRI scan | Page 6 |
| k-space traversal | MRI | 25 | Describe the acquisition trajectory of the k-space. | NA |
| Number of averages/ excitations | MRI | 26 | Describe the number of times each point in k-space is sampled. | NA |
| Magnetic field strength | MRI | 27 | Describe the nominal strength of the MR magnetic field. | NA |
| Reconstruction |  |  |  |  |
| In-plane resolution | MRI | 28 | Describe the distance between pixels, or alternatively the field of view and matrix size | Page 6 |
| Image slice thickness | MRI | 29 | Describe the slice thickness | Page 6 |
| Image slice spacing | MRI | 30 | Describe the distance between image slices | Page 6 |
| Convolution kernel | CT | 31a | Describe the convolution kernel used to reconstruct the image. | NA |
|  | CT | 31b | Describe settings pertaining to iterative reconstruction algorithms. | NA |
| Exposure | CT | 31c | Describe the exposure (in mAs) in slices containing the region of interest. | NA |
| Reconstruction method | PET | 32a | Describe which reconstruction method was used | NA |
|  | PET | 32b | Describe the number of iterations for iterative reconstruction | NA |
|  | PET | 32c | Describe the number of subsets for iterative reconstruction. | NA |
| Point spread function modelling | PET | 33 | Describe if and how point-spread function modelling was performed. | NA |
| Image corrections | PET | 34a | Describe if and how attenuation correction was performed. | NA |
|  | PET | 34b | Describe if and how other forms of correction were performed | NA |
| Reconstruction method | MRI | 35a | Describe the reconstruction method used to reconstruct the image from the k-space information. | NA |
|  |  | 35b | Describe any artifact suppression methods used during reconstruction to suppress artifacts due to undersampling of k-space | NA |
| Diffusion-weigh ted imaging | DWI-MR | 36 | Describe the b-values used for diffusion-weigh ting. | Page 6 |
| Image registration |  |  |  |  |
| Registration method |  | 37 | Describe the method used to register multi-modality imaging. | NA |
| Data conversion |  |  |  |  |
| SUV normalisation | PET | 38 | Describe which standardised uptake value (SUV) normalisation method is used. | NA |
| ADC computation | MRI | 39 | Describe how apparent diffusion coefficient (ADC) values were calculated. | Page 7-8 |
| Other data conversions |  | 40 | Describe any other conversions that are performed to generate. | NA |
| Post-acquisition processing |  |  |  |  |
| Anti-aliasing |  | 41 | Describe the method used to deal with anti-aliasing when down-sampling during interpolation. | NA |
| Noise suppression |  | 42 | Describe methods used to suppress image noise | NA |
| Post-reconstruc tion smoothing filter | PET | 43 | Describe the width of the Gaussian filter (FWHM) to spatially smooth intensities. | NA |
| Skull stripping | MRI (brain) | 44 | Describe method used to perform skull stripping. | NA |
| Non-uniformity correction | MRI | 45 | Describe the method and settings used to perform non-uniformity correction. | NA |
| Intensity normalisation |  | 46 | Describe the method and settings used to normalise intensity distributions within a patient or patient cohort. | NA |
| Other post-acquisitio n processing methods |  | 47 | Describe any other methods that were used to process the image and are not mentioned separately in this list. | NA |
| Segmentation |  |  |  |  |
| Segmentation method |  | 48a | Describe how regions of interest were segmented | Page 7-8 |
|  |  | 48b | Describe the number of experts, their expertise and consensus strategies for manual delineation. | Page 7-8 |
|  |  | 48c | Describe methods and settings used for semi-automatic and fully automatic segmentation | Page 7-8 |
|  |  | 48d | Describe which image was used to define segmentation in case of multi-modality imaging. | Page 7-8 |
| Conversion to mask |  | 49 | Describe the method used to convert polygonal or mesh-based segmentations to a voxel-based mask. | The ROI masks are directly stored as voxels in the DICOM Segmentation image format, and the ROI masks are generated by loading corresponding images. |
| Image interpolation |  |  |  |  |
| Interpolation method |  | 50a | Describe which interpolation algorithm was used to interpolate the image. | The nearest neighbour algorithm was used for interpolation |
|  |  | 50b | Describe how the position of the interpolation grid was defined. | Align grid centers |
|  |  | 50c | Describe how the dimensions of the interpolation grid were defined. | Rounded to nearest integer |
|  |  | 50d | Describe how extrapolation beyond the original image was handled | The image should be sufficiently padded with voxels that take on the nearest boundary intensity. |
| T |  | 51 | Describe the size of the interpolated voxels. | 3.0×3.0×3.0 mm^3^ |
| Intensity rounding | CT | 52 | Describe how fractional Hounsfield Units are rounded to integer values after interpolation. | NA |
| ROI interpolation |  |  |  |  |
| Interpolation method |  | 53 | Describe which interpolation algorithm was used to interpolate the region of interest mask. | The nearest neighbour algorithm |
| Partially masked voxels |  | 54 | Describe how partially masked voxels after interpolation are handled | NA |
| Re-segmentation |  |  |  |  |
| Re-segmentation methods |  | 55 | Describe which methods and settings are used to re-segment the ROI intensity mask. | Intensity outlier filtering |
| Discretisation |  |  |  |  |
| Discretisation method |  | 56a | Describe the method used to discretise image intensities. | Fixed bin number |
|  |  | 56b | Describe the number of bins (FBN) or the bin size (FBS) used for discretisation | 32 bins |
|  |  | 56c | Describe the lowest intensity in the first bin for FBS discretisation | NA |
| Image transformation |  |  |  |  |
| Image filter |  | 57 | Describe the methods and settings used to filter images. | Laplacian-of-Ga ussian |
|  |  |  |  |  |
| Image biomarker computation |  |  |  |  |
| Biomarker set |  | 58 | Describe which set of image biomarkers is computed and refer to their definitions or provide these. | Supplementary Method S1 |
| IBSI compliance |  | 59 | State if the software used to extract the set of image biomarkers is compliant with the IBSI benchmarks. | Page 8 |
| Robustness |  | 60 | Describe how robustness of the image biomarkers was assessed. | Test-retest analysis. |
| Software availability |  | 61 | Describe which software and version was used to compute image biomarkers. | Page 8 |
| Texture parameters |  |  |  |  |
| Texture matrix aggregation |  | 62 | Define how texture-matrix based biomarkers were computed from underlying texture matrices. | GLCM, GLRLM: BTW3  GLSZM, GLDZM, NGTDM, NGLDM: 8QNN |
| Distance weighting |  | 63 | Define how CM, RLM, NGTDM and NGLDM weight distances. | No weighting. |
| CM symmetry |  | 64 | Define whether symmetric or asymmetric co-occurrence matrices were computed. | symmetric |
| CM distance |  | 65 | Define the (Chebyshev) distance at which co-occurrence of intensities is determined1. | Chebyshev distance of 1 |
| SZM linkage distance |  | 66 | Define the distance and distance norm for which voxels with the same intensity are considered to belong to the same zone for the purpose of constructing an SZM. | Chebyshev distance of 1 |
| DZM linkage distance |  | 67 | Define the distance and distance norm for which voxels with the same intensity are considered to belong to the same zone for the purpose of constructing a DZM. | Chebyshev distance of 1 |
| DZM zone distance norm |  | 68 | Define the distance norm for determining the distance of zones to the border of the ROI. | Manhattan distance |
| NGTDM distance |  | 69 | Define the neighbourhood distance and distance norm for the NGTDM, e.g.. | Chebyshev distance of 1 |
| NGLDM distance |  | 70 | Define the neighbourhood distance and distance norm for the NGLDM,. | Chebyshev distance of 1 |
| NGLDM coarseness |  | 71 | Define the coarseness parameter for the NGLDM. | 0 |
| Machine learning and radiomics analysis |  | 72 |  |  |
| Diagnostic and prognostic modelling |  | 73 | See the TRIPOD guidelines for reporting on diagnostic and prognostic modelling. | Page 13 |
| Comparison with known factors |  | 74 | Describe where performance of radiomics models is compared with known (clinical) factors. | NA |
| Multicollineari ty |  | 75 | Describe where the multicollinearity between image biomarkers in the signature is assessed. | Page 12, Supplementary Methods 2 |
| Model availability |  | 76 | Describe where radiomics models with the necessary pre-processing information may be found. | NA |
| Data availability |  | 77 | Describe where imaging data and relevant meta-data used in the study may be found. | Page 24 |

***** pages in manuscript; NA: not application
